# Club-based vs. hospital-guided rehabilitation after ACL reconstruction in football participants: a two-year prospective cohort study

**DOI:** 10.3389/fspor.2025.1641750

**Published:** 2025-09-02

**Authors:** Zhe Han, Peng Sun, Tianwu Chen, Yu Dong

**Affiliations:** ^1^ Department of Physical Education, Dongbei University of Finance and Economics, Dalian, China; ^2^ Sports Performance Unit, Dalian Professional Football Club, Dalian, China; ^3^ Sports Performance Unit, Dalian Yingbo Football Club, Dalian, China; ^4^ Department of Sports Medicine, Huashan Hospital, Fudan University, Shanghai, China

**Keywords:** anterior cruciate ligament, football, rehabilitation, club-based, hospital-guided

## Abstract

**Background:**

Anterior cruciate ligament (ACL) injury is a common and serious sports injury in football. Although reconstruction surgery can restore joint stability, postoperative rehabilitation strategies play a critical role in long-term outcomes, particularly return to sport (RTS). However, comparative studies on the long-term efficacy of hospital-guided vs. club-based rehabilitation programs remain limited.

**Purpose:**

To compare the two-year clinical outcomes and RTS of hospital-guided and club-based rehabilitation programs following ACL reconstruction in football participants.

**Methods:**

This prospective cohort study enrolled 40 football participants who underwent primary ACL reconstruction. Participants were assigned to two groups based on rehabilitation modality: hospital-guided rehabilitation (*n* = 20), which emphasized basic joint function recovery, and club-based rehabilitation (*n* = 20), which focused on sport-specific physical conditioning and performance enhancement. Outcome measures included subjective functional scores: Tegner Activity Score (TAS), Marx Activity Rating Scale (MARS), International Knee Documentation Committee (IKDC) subjective score, Lysholm Knee Rating Scale (LKSS), and Anterior Cruciate Ligament-Return to Sport after Injury (ACL-RSI), as well as objective knee function [effusion, range of motion (ROM), and joint stability], time to return to training and competition, and re-injury rate.

**Results:**

The two groups were comparable at baseline. The club-based group demonstrated significantly better outcomes in TAS (8.5 ± 1.4 vs. 7.2 ± 1.0, *p* = 0.006), MARS (12.7 ± 2.8 vs. 10.6 ± 2.7, *p* = 0.019), and ACL-RSI (73.3 ± 21.4 vs. 54.3 ± 18.7, *p* = 0.003). Time to return to training and competitive play was significantly shorter in the club-based group. No significant differences were observed in ROM, joint stability, or re-injury rate between groups.

**Conclusion:**

Club-based rehabilitation led to superior subjective function, greater psychological readiness, and earlier return to sport compared to hospital-guided programs, without increasing re-injury risk. These findings support the effectiveness of sport-specific rehabilitation in football players after ACL reconstruction, though further studies are needed to evaluate long-term safety and cost-effectiveness.

## 1 Introduction

Anterior cruciate ligament (ACL) injury is one of the most common and career-threatening injuries in football, severely impairing athletic performance and long-term participation (1). Although ACL reconstruction has become the standard surgical procedure to restore knee joint stability, the postoperative rehabilitation process plays an equally critical role in regaining functional capacity and ensuring a safe return to play (2, 3). In recent years, the focus of sports medicine has gradually shifted from mere “structural reconstruction” to “functional rehabilitation”, highlighting the need for sport-specific and individualized rehabilitation strategies that align with the athletic demands of the target population (4).

Currently, rehabilitation models following ACL reconstruction can be broadly categorized into three types: home-based self-training, hospital-guided rehabilitation, and club-based sport-specific rehabilitation (5–7). Among these, hospital-guided rehabilitation is typically led by orthopaedic sports medicine physicians and physical therapists, emphasizing pain control, joint range of motion (ROM), and muscle strength recovery (5). This approach is characterized by a high level of standardization and safety. However, it often falls short in restoring sport-specific capabilities such as explosiveness, agility, dynamic balance, and tolerance to high-intensity competition: factors that are critical for elite football players aiming to return to play.

In contrast, club-based rehabilitation is usually conducted in collaboration between sports rehabilitation specialists and strength & conditioning coaches (8). It incorporates sport-specific simulation training, functional assessments, and psychological readiness evaluations, closely mimicking real-game scenarios and promoting comprehensive athletic recovery (9). However, there remains a lack of high-quality prospective studies, particularly in football-specific cohorts. Notably, the two-year mark represents a critical timepoint after athletes have typically completed a full competitive cycle, offering a more realistic reflection of functional recovery and re-injury risk, and thereby a better measure of the long-term effectiveness of rehabilitation strategies (10).

Therefore, this prospective cohort study aims to systematically compare the two-year outcomes of hospital-guided vs. club-based rehabilitation programs in football athletes following ACL reconstruction, focusing on:

(1) subjective symptom improvement and joint function scores,
(2) restoration of sport-specific performance,
(3) psychological readiness, and
(4) return-to-training and return-to-competition timing and rates, as well as re-injury incidence.

We hypothesize that compared with hospital-guided rehabilitation, club-based sport-specific rehabilitation will significantly enhance sport-specific functional recovery, athletic performance, and psychological confidence in football players after ACL reconstruction, without increasing the risk of re-injury. Consequently, this study seeks to fill the evidence gap regarding long-term rehabilitation efficacy in sport-specific populations, providing clinically actionable recommendations. Additionally, our findings may inform future stratified rehabilitation planning, resource allocation, and cost-effectiveness analysis in the field of sports medicine. The insights gained from this study may also be generalizable to other high-demand sports with similar biomechanical characteristics, such as basketball and rugby, offering broad international relevance and translational potential.

## 2 Methods

### 2.1 Study design and participants

This study adopted a prospective cohort design. Participants were recruited from football players and football enthusiasts who underwent ACL reconstruction between 2018 and 2021 at the Department of Sports Medicine, Huashan Hospital, Fudan University. All included patients had engaged in regular football activities prior to surgery. Professional football players were defined as those receiving systematic football training and participating in training or competition at least five times per week. Football enthusiasts were defined as individuals without a professional background who participated in football training or matches at least twice per week. All surgical procedures were performed by Dr. Yu Dong and Tianwu Chen.

Based on postoperative rehabilitation modality, patients were assigned to either a hospital-guided rehabilitation group or a club-based rehabilitation group. Patients in the hospital group underwent a function-oriented rehabilitation program at Huashan Hospital's Sports Medicine Center. Patients in the club group received a sport-specific rehabilitation program led by Coach Peng Sun and his team at the Dalian Football Base, focusing on the restoration of football-related performance.

### 2.2 Inclusion and exclusion criteria and sample size calculation

Inclusion criteria:

(1) Football players or enthusiasts aged 18–40 years;
(2) Isolated ACL injury or ACL injury combined with meniscal damage;
(3) Underwent primary ACL reconstruction at the Sports Medicine Department, Huashan Hospital;
(4) Graft type was either autologous hamstring tendon or synthetic ligament;
(5) Completed the full rehabilitation protocol and had complete 2-year follow-up data;
(6) No history of other major lower limb injuries prior to surgery.

Exclusion criteria:

(1) Multiligamentous injuries or severe cartilage damage;
(2) History of other lower limb surgeries or severe chronic diseases;
(3) Change of rehabilitation program or failure to complete scheduled follow-up;
(4) Serious postoperative complications (e.g., infection, thrombosis);
(5) Inability to comply with rehabilitation training or follow-up assessments.

The primary outcome for sample size estimation was time to return to sport. Preliminary pilot data indicated a significant difference in this variable between the club-based and hospital-guided rehabilitation groups. Assuming a two-sided test with an alpha level of 0.05% and 80% statistical power, the required sample size was calculated to be at least 16 participants per group. To account for potential loss to follow-up, the final sample size was set at 20 participants per group, totaling 40 patients, thereby ensuring sufficient statistical power to compare the effects of the two rehabilitation modalities on return-to-sport timing.

### 2.3 Postoperative rehabilitation and patient grouping

Based on the type of postoperative rehabilitation, patients were divided into two groups: the hospital-guided rehabilitation group and the club-based rehabilitation group. Both rehabilitation models incorporated the core components of standard postoperative ACL rehabilitation, including restoration of joint range of motion (ROM), control of swelling and pain, strength training for major muscle groups such as the quadriceps and hamstrings, gait correction, and balance and coordination exercises. In addition, psychological status and interventions were also considered important in both programs.

The hospital-guided rehabilitation program was conducted at the Sports Medicine Center of Huashan Hospital, Fudan University. It was supervised jointly by orthopedic surgeons and physical therapists, with the frequency and content of training progressing in accordance with standardized protocols. This approach emphasized safety and standardization in the process of functional recovery. More details were presented in Tables 1A,B.

**Table 1A:** Comparative rehabilitation protocol for autograft ACL reconstruction: hospital-guided vs. club-based programs.

Phase	Rehabilitation goals	Hospital-guided program	Club-based program	Comparison analysis
Acute phase (0–2 weeks)	Control swelling and pain, restore early ROM, activate muscle contraction	- Intermittent cryotherapy and elastic compression bandage for postoperative swelling - ROM training (0–90°) via CPM, passive flexion, self-assisted stretching - Isometric quadriceps contraction - Limb elevation and ankle pumps to prevent DVT - Non-weight-bearing ambulation with crutches, emphasizing proper gait mechanics	- Same goals as hospital, but led by active training - NMES-assisted quadriceps activation - Trunk stabilization exercises: incline bridge, side plank - Hip abduction/external rotation and ankle stabilization drills - Core initiation and posture control on balance pads	Shared goals with slightly different approaches. Hospital prioritizes passive and safe methods, while club emphasizes proactive activation and neuromuscular stimulation.
Early phase (2–6 weeks)	Progressively improve ROM, re-establish gait pattern, strengthen primary muscles and proprioception	- Increase ROM to 120°, mobilize peripatellar soft tissues - Gait training progresses from double to single crutch, focusing on hip-knee coordination - Resistance training for quadriceps, hamstrings, glutes: SLR, resistance band extensions - Balance drills on stable surfaces with eyes closed and perturbationBasic core activation	- Dynamic stretching + fascial release assist ROM - Gait retraining with floor tape, mirror feedback, manual cueing - Hip-knee chain coordination exercises - Balance/sensory-motor integration: Bosu ball, blindfolded catch, wobble board - Introduce variable-speed walking and quick stops	Similar strategies for ROM and gait; club program enhances neuromotor synergy and proprioceptive input for improved movement quality.
Mid phase (6 weeks–4 months)	Establish weight-bearing tolerance, develop unilateral lower limb stability, initiate early sport-specific elements	- Restore ROM to contralateral level (>135°), pass deep squat and kneeling test - Full weight-bearing gait with correction via video analysis - Strengthening: double-leg squats, wall sits, lunges, step-ups - Single-leg balance training with foam and balance boards - Core stabilization: bridges, side planks, planks - No running/jumping at this stage, focus on joint stability	- Unilateral resistance training: Romanian deadlifts, side plank + leg raises - ROM >135°, maintained with foam rolling and dynamic mobility - Dynamic balance: Bosu transitions, blindfolded catch, Y-balance patterns - Cardiovascular training via bike or elliptical, avoiding impact - Low-intensity ball drills: static passing/receiving - Emphasis on control and endurance	Both focus on strength and balance. Hospital ensures safety and standardization; club enhances motor precision and builds early foundations for sport-specific return.
Late phase (4–6 months)	Improve sport-specific capacity, enhance jumping/cutting ability, simulate match situations	- Unilateral jumping and directional control: single-leg hops, lateral hops, scissors jumps - Agility: ladder drills, cone slalom, start/stop drills - Advanced strength: barbell squats, leg press, kettlebell work - Landing mechanics instruction: prevent valgus/overextension - Psychological interview and ACL-RSI assessment	- Agility/cutting: 5-10-5 shuttle, arrow run, blindfolded slalom - Controlled contact drills: 1v1 possession, targeted passing - FIFA11 + core integration: balance lunges, dynamic mobility, trunk control - Video feedback on jumping/landing mechanics with repeated practice	Hospital focuses on functional recovery; club program intensifies variability and realism, simulating real match environments to build confidence and reactivity.
Return-to-sport phase (6–12 months)	Optimize movement patterns, strengthen psychological readiness, enable safe and high-quality return	- Jump-landing retraining via video feedback - Functional assessments: SL hop, *T*-test, Y-balance - Psychological interventions: goal-setting, graduated exposure, scenario simulation - Regular knee stability evaluations to assess readiness - Full return to competitive matches typically recommended at 12 months	- Phased match simulations: 3v3 half-field, possession drills - Video-assisted technical correction - ACL-RSI and sports confidence questionnaires - Planned reintroduction to low-risk warm-up matches	Shared goals. Hospital ensures safe and standardized clearance; club promotes match integration with FIFA11 + modules and psychological profiling, offering a more systematic return.

**Table 1B:** Comparative rehabilitation protocol for ACL reconstruction using artificial ligament: hospital-guided vs. club-based programs.

Phase	Rehabilitation goals	Hospital-guided program	Club-based program	Comparison analysis
Initial recovery (0–2 weeks)	- Control postoperative inflammation and pain - Protect incision and initiate early ROM - Activate quadriceps to prevent muscle inhibition	- Intermittent cryotherapy, elastic compression - ROM 0–90° via CPM, passive flexion, self-stretching - Isometric quadriceps contraction (daily multiple sets) - Limb elevation and ankle pumps to prevent DVT - Gait training with bilateral crutches, non-weight-bearing	- Same as hospital, with greater focus on active participation - NMES-assisted quadriceps activation - Core stabilization (bridge, side plank) - Hip/ankle neuromuscular training - Balance pad for posture and proprioception	Shared objectives. Club emphasizes more proactive motor engagement and neuromuscular activation, while hospital prioritizes safe, standardized protocols
Functional enhancement (2–6 weeks)	- Achieve 120–130° ROM - Regain independent walking with normalized gait - Strengthen quadriceps, hamstrings, glutes - Improve proprioception and core control	- Active ROM training, peripatellar mobilization - Transition to single crutch walking, gait correction - Resistance training (SLR, band extension/flexion) - Bilateral balance training on stable platforms - Basic core activation	- Dynamic stretching + foam rolling for ROM - Gait pattern correction with visual and manual cues - Coordinated hip–knee chain training - Advanced proprioceptive drills: Bosu, closed-eye catch - Variable-speed walking, quick-stop practice	Similar structure, club emphasizes neuromuscular coordination, faster dynamic control integration, and sensory-cognitive complexity
Functional consolidation & sports transition (7–12 weeks)	- Achieve full ROM (>135°) - Strength symmetry ≥95% of contralateral side - Master movement patterns and stability - Introduce running, jumping, sport-specific drills	- Double-leg and single-leg closed kinetic chain exercises (squats, lunges, step-ups) - Dynamic balance on foam, wobble boards - Bridge series, planks for trunk stability - Controlled introduction of double-leg hops and agility drills - Video feedback on movement control	- Unilateral strengthening: TRX squats, RDLs - Landing drills - Low-intensity ball drills: short-pass, static receive - FIFA11 + core elements - Small-group simulation games after 12 weeks - If meniscus repair, delay jumping/running to 16 weeks	Hospital focuses on structural restoration and safety. Club prioritizes sport readiness and realistic scenarios with earlier running/jumping (from 4 weeks), unless restricted by meniscal repair. Greater variability and progression with sport-specific content

ROM, range of motion; CPM, continuous passive motion; NMES, neuromuscular electrical stimulation; DVT, deep vein thrombosis; SLR, straight leg raise; TRX, suspension training system; RDL, Romanian deadlift.

The club-based rehabilitation program was implemented at the Dalian Football Base under the guidance of Coach Peng Sun and his team. After completing the foundational rehabilitation components, participants received intensive, football-specific training, including high-level tasks such as agility drills, explosive power training, complex change-of-direction maneuvers, passing and receiving exercises, and simulated contact scenarios. This rehabilitation model involved higher training frequency and engagement, with most participants performing two sport-specific sessions per day. The intensity and time commitment were substantially greater than those of the hospital-guided group, aiming to more effectively restore football players and enthusiasts to their pre-injury competitive level and support a safe and high-quality return to sport.

### 2.4 Follow-up assessment

This study included two assessment time points: a preoperative baseline evaluation and a two-year postoperative follow-up. The evaluations covered three main domains: subjective function, objective clinical examination, and return-to-sport outcomes.

Subjective functional assessment utilized a range of internationally recognized scoring tools, including the International Knee Documentation Committee (IKDC) subjective score, the Lysholm Knee Scoring Scale (LKSS), the Tegner Activity Score, the Marx Activity Rating Scale, and the Anterior Cruciate Ligament–Return to Sport after Injury (ACL-RSI) scale. These instruments were used to comprehensively evaluate patients' perceptions of knee function, pain, athletic capacity, and psychological readiness.

Objective clinical measures included assessments of knee joint swelling, stability, and range of motion. Swelling was evaluated by visual inspection and palpation. Stability was assessed via the Lachman test and pivot-shift test. Range of motion was measured based on knee flexion and extension angles.

Return-to-sport outcomes included the timing of the patient's first return to structured football training or participation in recreational/formal matches (i.e., return-to-sport timing), the postoperative athletic level achieved (as analyzed by comparing the Tegner score to the pre-injury level), and adverse events. The latter included major complications such as infection, hematoma, joint stiffness, recurrent laxity, and whether reinjury occurred within two years (including ACL rerupture or other significant lower extremity sports-related injuries). These parameters collectively provided a comprehensive evaluation of rehabilitation outcomes and long-term sports function recovery.

### 2.5 Statistical analysis

Data analysis was performed using SPSS or R statistical software. Continuous variables (e.g., IKDC score, Lysholm score, Tegner score, Marx score, and ACL-RSI score) were first tested for normality. Variables following a normal distribution were compared between groups using independent-samples *t*-tests, while those not conforming to normal distribution were analyzed using the Mann–Whitney *U* test. Categorical variables (e.g., return-to-sport rates, reinjury incidence) were analyzed using the chi-square test to assess differences between groups. A significance level of *α* = 0.05 was set for all statistical tests.

### 2.6 Ethical approval

The study protocol was approved by the Ethics Committee of Huashan Hospital, Fudan University. Informed consent was obtained from all participants.

## 3 Results

### 3.1 Baseline data comparison

The two patient groups demonstrated good comparability in their preoperative baseline characteristics. Specifically, there were no statistically significant differences (all *P* > 0.05) between the hospital-guided rehabilitation group and the club-based rehabilitation group in terms of age, sex distribution, preoperative Tegner activity scores, proportion of professional football players, dominance of the operated limb (dominant vs. non-dominant), graft type (autologous hamstring tendon or artificial ligament), and the proportion of concomitant meniscal injuries. Additionally, no significant differences were observed in preoperative Lysholm scores, IKDC scores, or ACL-RSI scores, indicating similar subjective functional status and psychological readiness across both groups before surgery. These findings suggest good baseline equivalence between the two groups, providing a solid foundation for comparing postoperative outcomes associated with different rehabilitation approaches. All patients completed the two-year postoperative evaluation. Detailed demographic and baseline clinical data are presented in Table 2.

**Table 2:** Demographic and baseline evaluation data.

Variable	Club-based group (*n* = 20)	Hospital-guided group (*n* = 20)	*P*-value
Age (years, mean ± SD)	28.3 ± 6.3	27.5 ± 6.9	0.704
Professional football players [*n* (%)]	11 (55%)	6 (30%)	0.110
Surgical side (Left/Right)	9/11	11/9	0.527
Graft type (Artificial ligament/Autologous tendon)	13/7	11/9	0.519
Concomitant meniscal injury [*n* (%)]	12 (60%)	13 (65%)	0.744
Concomitant cartilage injury [*n* (%)]	7 (35%)	5 (25%)	0.490
Preoperative Tegner score (mean ± SD)	2.1 ± 1.4	2.4 ± 1.5	0.533
Preoperative Lysholm score (mean ± SD)	50.9 ± 12.7	42.9 ± 22.8	0.448
Preoperative IKDC score (mean ± SD)	37.9 ± 13.8	31.7 ± 15.8	0.172
Preoperative ACL-RSI score (mean ± SD)	34.4 ± 20.3	30.9 ± 17.9	0.636

### 3.2 Subjective functional outcomes

There were significant differences in the Tegner, Marx, and ACL-RSI scores between the two groups, with the club-based rehabilitation group scoring significantly higher than the hospital-guided group. However, no significant differences were observed in the IKDC and Lysholm scores. More details are provided in Table 3.

**Table 3:** Subjective functional outcomes at two-year follow-up.

Subjective score	Club-based group (*n* = 20)	Hospital-guided group (*n* = 20)	*P*-value
Tegner score (mean ± SD)	8.5 ± 1.4	7.2 ± 1.0	0.006
Marx score (mean ± SD)	12.7 ± 2.8	10.6 ± 2.7	0.019
Lysholm score (mean ± SD)	93.2 ± 8.3	90.2 ± 9.4	0.367
IKDC score (mean ± SD)	90.0 ± 11.1	91.5 ± 8.6	0.913
ACL-RSI score (mean ± SD)	73.3 ± 21.4	54.3 ± 18.7	0.003

### 3.3 Objective clinical assessments

In terms of objective indicators, there were no significant differences between the two groups in joint swelling, stability, or range of motion at the two-year follow-up (see Table 4).

**Table 4:** Objective clinical assessments at two-year follow-Up.

Clinical examination	Club-based group (*n* = 20)	Hospital-guided group (*n* = 20)	*P*-value
Evaluation grade	A/B/C/D	A/B/C/D	—
Joint swelling	18/2/0/0	20/0/0/0	0.147
Extension limitation	20/0/0/0	20/0/0/0	0.999
Flexion limitation	20/0/0/0	19/1/0/0	0.311
Lachman test	18/2/0/0	17/3/0/0	0.633
Anterior drawer test	19/1/0/0	18/2/0/0	0.548
Lever test	20/0/0/0	20/0/0/0	0.999

### 3.4 Return-to-Sport outcomes

All patients in both groups successfully returned to recreational football. For competitive football, the return-to-sport rate at 2 years postoperatively was 90% in the club-based rehabilitation group and 65% in the hospital-guided group. In terms of return timing, the club-based group resumed football training games (recreational matches) and official matches significantly earlier than the hospital-guided group (see Table 5).

**Table 5:** Return-to-sport data at two-year follow-up.

Type of return to sport	Club-based group (*n* = 20)	Hospital-guided group (*n* = 20)	*P*-value
Recreational match/training game (weeks)	12.4	18.3	0.042
Official match (weeks)	19.0	26.5	0.002

## 4 Adverse events and follow-up compliance

No serious complications occurred in either group during follow-up. One case of reinjury was reported in each group. In the club-based group, one patient experienced loosening of tibial fixation and a medial meniscus injury, which was managed with arthroscopic surgery. In the hospital group, one patient sustained a medial collateral ligament (MCL) sprain, which was treated conservatively with full recovery and return to football activities.

## 5 Discussion

This study provides compelling evidence that club-based, sport-specific rehabilitation leads to superior two-year outcomes in football players following ACL reconstruction, particularly in subjective functional recovery and RTS parameters. Compared to the traditional hospital-guided model, the club-based approach significantly improved Tegner, Marx, and ACL-RSI scores and enabled an earlier return to both recreational and competitive football, highlighting its clinical value in restoring performance and psychological readiness.

These findings align with previous work suggesting that sport-specific and high-intensity rehabilitation programs can better address the complex neuromuscular demands of return to football (11, 12). The present study extends this evidence by providing a two-year follow-up in a real-world football cohort, which is often lacking in the literature.

A key strength of this study is its pragmatic, real-world design. By including both professional players and football enthusiasts, as well as patients with different graft types (autologous hamstring tendon and synthetic ligament), the cohort reflects the actual heterogeneity seen in clinical practice. This inclusiveness enhances external validity and generalizability of the findings to diverse football populations. However, it also introduces potential confounding factors that must be acknowledged. For example, professional players may have better baseline physical conditioning, greater motivation, and more familiarity with structured training, all of which could influence outcomes independently of the rehabilitation modality (13, 14).

Although baseline characteristics were statistically balanced between groups, subtle unmeasured differences may remain. For instance, even though graft type (artificial ligament vs. autograft) was not significantly different between groups, surgical technique differences might influence rehabilitation trajectories (2, 3, 15). While the study controlled for major confounders via inclusion criteria and baseline matching, residual confounding is an inherent limitation of cohort designs. Future studies could further address this by stratifying analyses by player level and graft type, or by using statistical methods such as multivariate regression or propensity score adjustment.

Additionally, while the club-based rehabilitation model involved more intensive and sport-specific training that leads to earlier RTS, it also requires higher athlete commitment, greater time investment, and potentially higher costs. These factors may limit accessibility and scalability, particularly in resource-constrained settings. Nonetheless, these same features are integral to its effectiveness, as they simulate real match demands and promote psychological readiness for competition.

Importantly, the inclusion of both artificial ligament and autologous tendon reconstructions is not merely a confounding factor but also a strength of this dataset. It reflects a true “real-world” clinical setting where patients choose the graft most appropriate based on healthcare providers' suggestion (15, 16). Such diversity enhances the relevance of the findings to daily practice in sports medicine.

Despite these limitations, this study provides robust, clinically meaningful evidence supporting the superiority of club-based rehabilitation in enhancing functional recovery, sport readiness, and psychological resilience in football players after ACL reconstruction. Clinicians, physical therapists, and strength & conditioning coaches should consider integrating sport-specific elements into rehabilitation planning, while tailoring programs to individual needs and monitoring for potential risks such as overtraining or reinjury.

## 6 Conclusion

Compared to hospital-guided rehabilitation, club-based rehabilitation resulted in significantly better subjective functional scores and psychological readiness, and enabled an earlier return to sport over the two-year follow-up period. These findings suggest that sport-specific rehabilitation may be more effective in addressing the performance demands of football players after ACL reconstruction. However, as no differences were found in objective knee function, further studies are warranted to explore the long-term safety, cost-effectiveness, and individual suitability of each rehabilitation approach.

## Data Availability

The datasets presented in this article are not readily available because they are the property of the two corresponding authors. At the outset of the study, all participants were informed and consented that their clinical and rehabilitation-related data would be used exclusively for research purposes within the authors’ affiliated institution. In accordance with this agreement, the dataset is not publicly available and will not be shared with external parties. Requests to access the datasets should be directed to dongyu.dy@163.com.
